# Analysis and Distribution of Emergency Cases at One of Mexico’s Largest Hospitals and Trauma Centers

**DOI:** 10.7759/cureus.81204

**Published:** 2025-03-25

**Authors:** Marco A Hernandez-Guedea, Gabriel García-González, José A Alipi-García, Francisco G Kreitler-Mayer, Betsaida Rodríguez-Medina, Roberto Ruiz-Badillo, Williams Luciano López-Vidal, José F Islas-Cisneros, Gerardo R Padilla-Rivas, Mauricio M García-Pérez

**Affiliations:** 1 Emergency and Shock/Trauma Department, Hospital Universitario Dr. José Eleuterio González, Universidad Autónoma de Nuevo León, Monterrey, MEX; 2 Plastic and Reconstructive Surgery Department, Hospital Universitario Dr. José Eleuterio González, Universidad Autónoma de Nuevo León, Monterrey, MEX; 3 Emergency Department, Hospital Universitario Dr. José Eleuterio González, Universidad Autónoma de Nuevo León, Monterrey, MEX; 4 Biochemistry Department, Hospital Universitario Dr. José Eleuterio González, Universidad Autónoma de Nuevo León, Monterrey, MEX

**Keywords:** emergencies, major trauma center, politrauma, public health and safety, social and behavioral epidemiology

## Abstract

Objective

This study aims to identify the most common emergencies among the population in the metropolitan area of Monterrey, Mexico - one of the largest cities in the country - and compare these emergencies and their characteristics with those in other countries and geographical regions.

Methods

This cross-sectional study included all patients (n = 14,744) treated in the emergency department of Hospital Universitario Dr. José Eleuterio González between September and December 2023. The analysis encompassed all patients registered in the triage census by nurses, excluding those with incomplete records. Since all patients with sufficient data were included, sample size calculation was not required. Statistical analyses included the chi-square test and logistic regression for categorical variables.

Results

A total of 14,744 emergency department patients were reviewed. Among them, 54.7% were women, with a mean age of 39.07 years (±19.62). Of the total, 92% were from the Monterrey metropolitan area. Traumatic emergencies accounted for 27.5%, with limb injuries being the most common (10.5%). Nontraumatic emergencies made up 72.5%, with abdominal/pelvic pain being the most frequent complaint (13%). The internal medicine department received the highest number of patients (36.1%). The busiest day was Saturday (15.2%), with peak hours at 1:00 p.m. (5.8%).

Conclusions

The majority of patients were women aged 21-30 years from the metropolitan area, primarily presenting with nontraumatic conditions such as abdominal pain. The highest patient influx occurred on Saturdays at 1:00 p.m., with most cases managed by the internal medicine department. These findings may help authorities and clinicians optimize emergency care resources by anticipating the most common emergencies and peak demand times.

## Introduction

An urgency is a rapidly occurring situation that does not pose an immediate life-threatening risk but could become dangerous if not promptly addressed. In contrast, an emergency is a sudden condition that may result in immediate fatal outcomes if not quickly managed [1]. Therefore, the emergency department plays a crucial role in providing timely treatment for patients with acute or aggravated conditions, where immediate intervention is essential to prevent severe or fatal complications [1].

Over the past decade, there has been a global increase in emergency department visits [1]. At the extremes of life, males are more likely to seek emergency care, whereas females are more frequent users during adolescence and adulthood, with peak ages occurring at 0-4 years, 20-30 years, and over 80 years [2]. The most common emergencies involve respiratory illnesses (30%), gastrointestinal diseases (17%), and trauma (11%) [1,2].

Trauma is defined as a sudden injury to tissue caused by violence or an accident. It is classified into penetrating, blunt, and deceleration trauma. Traumatic injury is the leading cause of death worldwide. In the United States, it is the primary cause of death among young adults and accounts for 10% of all deaths in both men and women. Trauma results in 50 million emergency visits annually and constitutes one-third of intensive care unit admissions [3,4]. Falls and traffic accidents are responsible for up to 80% of traumatic injury cases requiring emergency care. The mortality rate for traumatic emergencies varies between 2.1% and 5.6%, depending on the medical center where the data was recorded [5]. Beyond its impact on health, trauma also has socioeconomic consequences, including loss of employment opportunities and financial instability [6].

The use of emergency services varies based on temporal and geographical factors. A study of two emergency departments in Berlin found the highest patient influx in spring and the lowest in autumn [7]. Similarly, research from a hospital in Sri Lanka reported peak emergency department admissions in March and April, with the busiest days being Fridays and Mondays [8,9]. Notably, a multicenter study in the United Kingdom found that adjusted odds of death for emergency admissions were 10% higher on weekends compared to weekdays [10]. Daily, the highest activity is typically observed between 8:00 a.m. and 12:00 p.m. [9], a pattern consistent across various healthcare centers [8].

Emergency department visits can be costly for patients compared to other forms of care. Rising healthcare expenses include both direct costs (such as medications and medical services) and indirect costs (such as transportation and lost work hours), which can significantly impact household financial stability, particularly when medical expenses exceed annual income [11]. The economic burden is even greater for patients with multimorbidity, who are often older [12]. By age 65, approximately 65% of individuals have multimorbidity, with prevalence being two to three times higher among lower socioeconomic populations [13]. Governments also face a substantial economic impact due to emergency care demands. In the United States, emergency services account for 5-12.5% of national health expenditures [14].

As of 2023, Mexico has a population of just over 131.1 million people. Of these, 19.5% are children aged 0-11 years, 30.7% are between 12 and 29 years, and more than half are over 30 years old. The country is predominantly urban, with 75% of its population residing in metropolitan areas and cities with at least 15,000 residents [15]. According to the 2020 National Institute of Statistics and Geography (INEGI) census, the male-to-female ratio was estimated at 95:100. Most of the population has completed primary education, with the remainder having secondary or higher education, while 4.9% have no formal education. Additionally, 98.1% of the population is economically active, with a slight predominance of males. In 2022, the general mortality rate in Mexico was 659 per 100,000 inhabitants, with leading causes of death including cardiovascular diseases, diabetes mellitus, malignant tumors, liver diseases, and COVID-19.

In 2020, INEGI reported that 73.5% of the population was affiliated with health services, with more than half being beneficiaries of the Mexican Social Security Institute (IMSS). In the private sector, there were 2,874 private healthcare establishments as of 2022. The most in-demand healthcare services in 2021 were specialty outpatient consultations, general medical consultations, and emergency services.

Monterrey, the capital of Nuevo León, is the center of the Monterrey Metropolitan Area, the second-largest metropolitan area in Mexico, with over five million inhabitants. As a major industrial, economic, and healthcare hub in northern Mexico, its hospitals serve not only local residents but also patients from neighboring states. The city has a population of 1,142,994, with a gender ratio of 97 men for every 100 women and a median age of 34 years. While basic education is predominant, higher education is more common in Monterrey compared to the rest of the country. According to the 2020 INEGI census, 79.2% of Monterrey’s population is affiliated with health services, mainly through IMSS.

Nuevo León has more than five tertiary care (specialty) hospitals. A significant portion of the state’s population seeks medical attention at Hospital Universitario Dr. José Eleuterio González, which, as of 2023, has the largest capacity in Mexico. The hospital has 1,244 beds - 700 for hospitalization, 194 for outpatient care, and 350 for multipurpose use - and is considered one of the best-equipped hospitals in the country [16].

Previous studies have compared another trauma center in Monterrey with a similar hospital in Seattle [17]. Understanding emergency department trends in this setting provides valuable insights into regional healthcare demands and resource allocation.

## Materials and methods

This study was conducted at Hospital Universitario Dr. José Eleuterio González in Monterrey, Nuevo León, located in northern Mexico. It is a descriptive observational cross-sectional study that recorded all patients who visited the triage area of the emergency department between September and December 2023. The objective was to analyze trends and identify patterns in consultation reasons, consultation timing, the service responsible for patient care, and patient sociodemographic characteristics.

Data were collected from the emergency department census, which is completed by nursing and medical staff during triage. One trained member from both the medical and nursing teams per shift was responsible for patient assessment and database entry, ensuring data consistency. The database was later updated with the final diagnosis assigned by the emergency physician or consulting specialist through the hospital’s internal system. The census included sociodemographic data (age, sex, and place of origin) and clinical data (admission diagnosis, department in charge, and admission date and time).

The sample size was determined by including all patients who visited the emergency triage area during the study period (September to December 2023). Given the observational and descriptive nature of the study, no prior sample size calculation was performed. Instead, a census approach was applied to ensure the inclusion of all eligible patients who met the established criteria.

Inclusion criteria encompassed all patients who sought care at the hospital’s emergency department. Exclusion criteria included patients without a recorded diagnosis by nursing or medical staff and those who left the hospital before their admission could be documented, ensuring that only complete medical assessments were analyzed.

This study was approved by the Research Ethics Committee of Hospital Universitario Dr. José Eleuterio González under registration code CP24-00001. Informed consent was obtained from all patients upon admission, as the University Hospital requires patient consent for education and research purposes.

Comprehensive statistical analysis was conducted using IBM SPSS Statistics for Windows, Version 25.0 (Released 2017; IBM Corp., Armonk, NY, USA). Data were initially reviewed for completeness and consistency, with missing values appropriately managed. The Kolmogorov-Smirnov test was used to assess the normality of continuous variables. Based on these results, either parametric or non-parametric tests were applied. Descriptive statistics were used to summarize data, with means and SDs reported for normally distributed continuous variables, while medians with interquartile ranges were used for non-normally distributed data.

Categorical variables were presented as frequencies and percentages. Associations between categorical variables were analyzed using Pearson’s chi-square test or Fisher’s exact test when expected cell counts were below five. To differentiate trauma from non-trauma admissions, comparisons were performed using independent t-tests or Mann-Whitney U tests, as appropriate.

To assess the strength of associations, ORs with 95% CIs were calculated where applicable. Multivariate analyses, including multinomial logistic regression, were conducted to control for potential confounders and identify independent predictors of outcomes. Admission diagnosis was chosen as the dependent variable, while sociodemographic variables were listed as independent variables in the model. Statistical significance was set at a two-tailed p-value ≤ 0.05 for all tests.

Graphs and tables were generated to visually represent the data, ensuring clarity in the distribution and trends observed. All analyses were carefully reviewed to ensure the robustness and reproducibility of findings.

## Results

**Figure 1:**
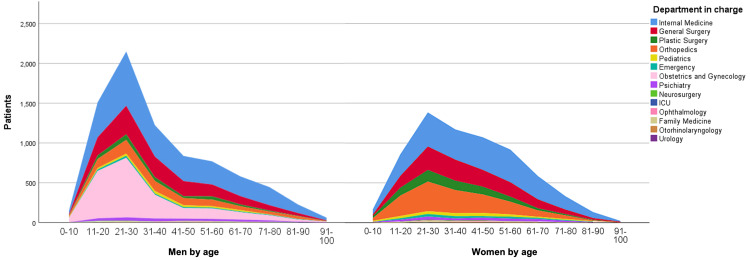
Patient distribution across hospital departments

During the four-month study period, from September 1, 2023, to December 31, 2023, a total of 14,744 emergency cases were recorded at the emergency department of Hospital Universitario Dr. José Eleuterio González. Among the patients treated, 54.7% were women. The average age was 39.07 years (±19.623), with the most represented age group being 21-30 years, accounting for 24% (3,541) of cases (Table 1, Figure 1). Additionally, 98 patients were excluded for not completing the triage process, as reported by security personnel.

**Table 1:** Sociodemographic characteristics, most common admission diagnoses, and peak admission days by age group

Age group	Gender (male/female)	Gender % (male/female)	Reason for consultation (%)	Day of the week (%)
0-10	169/149	53.1/46.9	Head and neck injury (9.7)	Thursday (20.4)
11-20	865/1,509	36.4/63.6	Pregnancy (19.6)	Monday (15.5)
21-30	1,392/2,149	39.3/60.7	Pregnancy (15.2)	Saturday (15.8)
31-40	1,183/1,224	49.1/50.9	Abdominal/pelvic pain (15.3)	Saturday (16.4)
41-50	1,085/837	56.5/43.5	Abdominal/pelvic pain (15.0)	Sunday (16.1)
51-60	934/769	54.8/45.2	Abdominal/pelvic pain (13.7)	Saturday (14.8)
61-70	599/578	50.9/49.1	Abdominal/pelvic pain (13.5)	Monday (15.7)
71-80	337/446	43.0/57.0	Abdominal/pelvic pain (11.7)	Friday (16.9)
81-90	138/227	37.8/62.2	Abdominal/pelvic pain (11.2)	Sunday (18.1)
91+	25/61	29.1/70.9	Head and neck injury (11.6)	Saturday (19.8)

Of the total patients served, 92% (13,573) were residents of the Monterrey Metropolitan Area, which includes the municipalities of Apodaca, Cadereyta Jiménez, El Carmen, García, San Pedro Garza García, General Escobedo, Guadalupe, Juárez, Monterrey, Salinas Victoria, San Nicolás de los Garza, Santa Catarina, and Santiago. Additionally, 3% (437) came from other municipalities within Nuevo León, 3.3% (479) from other states in Mexico, and 0.4% (58) from outside the country.

**Figure 2:**
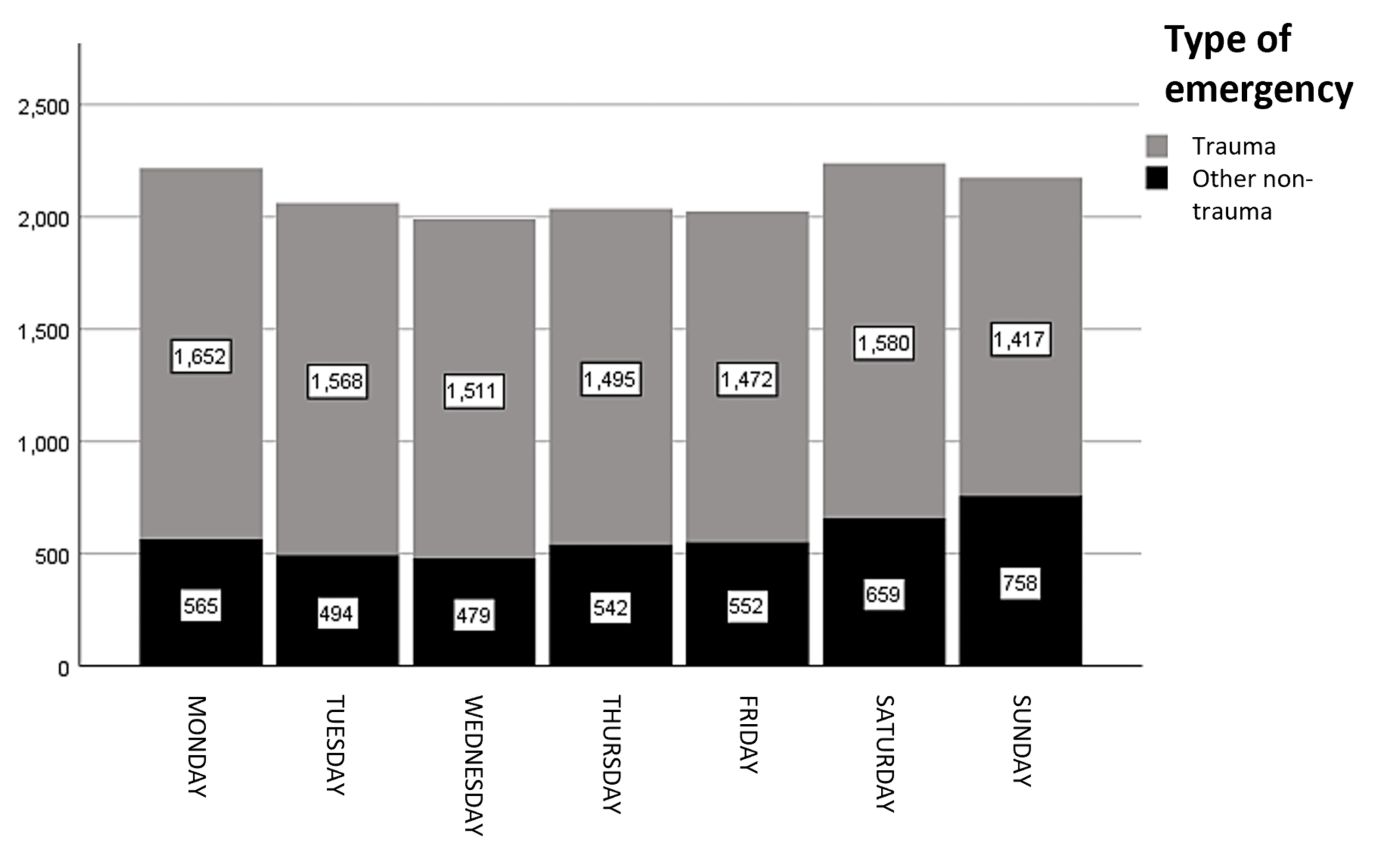
Distribution of traumatic vs. nontraumatic emergencies

Nontraumatic emergencies accounted for 72.5% of all cases (Figure 2). The most common reasons for consultation were abdominal/pelvic pain (13%, 1,919 cases), followed by other diagnoses (12.5%, 1,840), pregnancy-related cases (10.8%, 1,593), and dyspnea (4.5%, 666) (Table 2).

**Table 2:** Reasons for consultation ^a^ Time mode: the most frequent hour (in 24-hour format) at which the diagnosis occurs most often

Reason for consultation	Gender (male/female)	Gender % (male/female)	Median age	Time mode^a^	Day of the week (%)
Mental state alteration	260/284	47.8/52.2	44.98	16.00	Thursday (15.4)
Hypo/hyperglycemia	96/121	44.2/55.8	44.03	14.00	Monday and Thursday (17.1)
Blood pressure alteration	157/157	50.0/50.0	51.01	13.00	Thursday (18.5)
Fall	232/176	56.9/43.1	43.08	16.00	Tuesday (17.9)
Headache	127/95	57.2/42.8	42.89	16.00	Wednesday (18.9)
Seizure (epileptic)	64/65	49.6/50.4	45.23	10.00	Thursday (20.2)
Diarrhea	22/20	52.4/47.6	51.4	11.00	Monday (31)
Shortness of breath	309/357	46.4/53.6	46.37	12.00	Thursday (15.9)
Dysuria/anuria	71/66	51.8/48.2	40.46	12.00	Tuesday (19.6)
Abdominal/pelvic pain	732/1187	38.1/61.9	40.09	23.00	Monday (15.4)
Lumbar/renal pain	122/90	57.5/42.5	50.04	14.00	Saturday (18.4)
Chest pain	13/448	2.8/97.2	25.57	23.00	Saturday (17.1)
Edema	77/44	63.6/36.4	40.27	12.00	Saturday (18)
Pregnancy	0/1593	0.0/100.0	30.65	15.00	Monday (17.3)
Stroke	64/41	61.0/39.0	41.95	13.00	Tuesday (19)
Fever	82/112	42.3/57.7	56.08	20.00	Saturday (27.3)
Gastrointestinal bleeding	150/50	75.0/25.0	40.63	16.00	Saturday (25)
Knife wound	99/20	83.2/16.8	31.2	5.00	Sunday (31.9)
Gunshot wound	140/82	63.1/36.9	30.84	22.00	Sunday (20.2)
Suicide attempt/ideation	88/112	44.0/56.0	44.23	13.00	Monday and Wednesday (20)
Natural/drug intoxication	90/36	71.4/28.6	37.77	23.00	Thursday (22.2)
Neck and head injury	411/176	70.0/30.0	36.52	23.00	Sunday (19.4)
Limb injury	1072/475	69.3/30.7	37.28	17.00	Saturday (15.9)
Chest/abdomen/pelvis injury	68/44	60.7/39.3	38.85	10.00	Friday (25)
Dizziness/nausea	46/33	58.2/41.8	39.8	22.00	Monday (24.1)
Other	960/879	52.2/47.8	41.97	13.00	Monday (15.9)
Weakness/asthenia/adynamia	289/278	51.0/49.0	45.59	13.00	Monday (18.2)
Cardiac arrest	39/14	73.6/26.4	32.53	11.00	Wednesday and Friday (17)
Animal bite/sting	78/59	56.9/43.1	41.21	18.00	Tuesday and Sunday (17.5)
Polytrauma	669/244	73.3/26.7	33.61	22.00	Sunday (25.5)
Syncope	30/31	49.2/50.8	39.55	17.00	Wednesday and Sunday (18)
Vaginal bleeding	0/451	0.0/100.0	30.97	22.00	Wednesday (18)
Vomit/oral intolerance	102/138	42.5/57.5	39.4	13.00	Sunday (17.9)
Total	6759/7978	45.9/54.1	39.07	13.00	

The highest number of emergency cases occurred on Saturday, accounting for 15.2% (2,239) of the total, followed closely by Monday with 15% (2,217) of cases. When categorizing the days into working days (Monday to Friday) and nonworking days (Saturday and Sunday), 70.1% (10,330) of emergencies occurred on working days, while 29.9% (4,414) were recorded on nonworking days. Non-trauma emergencies consistently outnumbered trauma-related cases throughout the week. However, trauma cases showed a relative increase over the weekend, peaking on Sunday, whereas non-trauma cases remained relatively stable but declined slightly on Sunday. This trend suggests that work-related injuries are more common on weekdays, while trauma cases increase due to leisure activities on weekends (Figure 2). The distribution of emergencies throughout the day exhibited two peaks: the first at 1:00 p.m. (5.8%) and the second at 10:00 p.m. (5.7%).

Traumatic emergencies accounted for 27.5% of all cases (Figure 2). The most common reasons for consultation were extremity injuries at 10.5% (1,550), polytrauma at 6.2% (913), and neck and head injuries at 4% (587) (Table 2).

The emergency admissions were primarily handled by the following departments, ranked by the number of cases treated: internal medicine (36.1%, 5,318 cases), general surgery (18.6%, 2,744 cases), gynecology and obstetrics (15.2%, 2,240 cases), orthopedics (15%, 2,219 cases), and plastic surgery (5.9%, 864 cases) (Figure 1, Table 3).

**Table 3:** Percentage of care workload by department

Department	Patients	Percentage (%)
Internal medicine	5,318	36.1
General surgery	2,744	18.6
Plastic surgery	864	5.9
Orthopedics	2,219	15.1
Pediatrics	371	2.5
Emergency	263	1.8
Obstetrics and gynecology	2,240	15.2
Psychiatry	435	3.0
Neurosurgery	53	0.4
ICU	3	0.0
Ophthalmology	60	0.4
Family medicine	130	0.9
Otorhinolaryngology	21	0.1
Urology	23	0.2
Total	14,744	100.0

A Kolmogorov-Smirnov test was conducted to assess the normality of all variables. The results indicate that all tested variables significantly deviated from a normal distribution, with p-values of <0.001. Age, in particular, exhibited a moderate deviation from normality, while sex, service of admission, and origin displayed substantial deviations, reflecting categorical or skewed data patterns. Although date and time variables showed smaller deviations, they also failed to meet normality criteria.

A significant association was found between sociodemographic variables (age grouped by decades and sex) and factors such as reason for consultation, day of the week, time of arrival, and the department providing care (p < 0.001).

The Mann-Whitney U test was applied to compare emergency admissions based on trauma versus non-trauma cases and their distribution by age group. For admissions, the U statistic was 13,353,033.000, with a Z value of -36.107 and a p-value of <0.001, confirming a highly significant difference in diagnosis distribution between traumatic and nontraumatic cases, reflecting distinct emergency patterns. Regarding age groups, the Mann-Whitney U statistic was 19,628,287.000, with a Z value of -7.950 and a p-value of <0.001, indicating a statistically significant difference in age distribution. Patients with traumatic diagnoses were generally younger than those with nontraumatic diagnoses.

To evaluate the predictive power of analyzed variables in determining the reason for patient admission, a simple multinomial logistic regression was performed. The results indicate that sex and day of the week accounted for only 1.5% of the variance in admission reasons (adjusted R² = 0.015, F(14,734) = 115.764; p < 0.001). A slight increase in predictive power was observed for female sex (standardized β = 0.114; p < 0.001) and for admissions on Saturday and Sunday (standardized β = 0.047; p < 0.001).

## Discussion

One of the main strengths of this study is its large sample size (14,744 patients), which enhances the robustness of the findings and improves their generalizability to the region. Additionally, this is the first study to examine the distribution of emergency cases in a specific Mexican population, providing valuable baseline data for future research and healthcare policy planning.

The most frequent age group for emergency department visits was 21-30 years, a pattern consistent with studies on emergency admissions in other Mexican cities [18] and international locations such as the United Kingdom [19], the United States [20], and Spain [21,22]. This higher representation of young adults may be due to their active lifestyles and increased exposure to risks. A 2022 CDC report estimated that the emergency department visit rate for individuals aged 18-44 is 47 per 100 people, the third highest after elderly patients (over 75 years old) and infants under one year old [23].

Another significantly represented group in emergency visits is individuals over 65 years old [17,18,20]. However, in this study, their frequency was lower than in other settings. For instance, a study in Spain estimated that elderly patients account for 15-25% of emergency department consultations [24], similar to the 16% observed in our study. This discrepancy may be attributed to social and economic factors, as 37.9% of elderly individuals in Mexico lived in poverty in 2020, with 49.1% lacking education and 28.8% lacking social security. These factors may limit their access to emergency services [25]. Additionally, female patients had a higher emergency service usage rate, consistent with findings from studies in the United Kingdom, Spain, and the United States [19-21].

The three most common diagnoses in this study were abdominal/pelvic pain (13%), pregnancy (10.8%), and extremity injuries (10.5%). This contrasts with studies from Turkey, where the most frequent diagnoses were unspecified pain (24%), respiratory complaints (23.4%), and gastrointestinal complaints (18.7%) [22]. In Barcelona, injuries and poisoning (24%) were the most frequent, followed by abdominal, chest, and lumbar pain and fever (13.6%) and respiratory diseases (13.5%) [26]. Similarly, studies in the United Kingdom reported that respiratory, cardiovascular, and gastrointestinal diseases were the primary causes of emergency medical admissions, whereas extremity injuries, abdominal pain, and trauma were the leading causes of surgical emergencies [19]. A study in another Mexican city identified musculoskeletal (26%), digestive (26%), and respiratory (17%) conditions as the most common causes of emergency department visits [18].

Compared to other locations, Monterrey may benefit from healthcare policies focused on prenatal care, given that pregnancy-related emergencies were the second most common diagnosis, suggesting inadequate prenatal follow-up. Additionally, musculoskeletal and gastrointestinal complaints are prevalent, aligning with trends in other regions.

Emergency visit reasons varied significantly by age group. Head and neck injuries were most common among the youngest (0-10 years) and oldest (>91 years), likely due to pediatric falls and frailty-related trauma, respectively. Pregnancy-related visits predominated among adolescents and young adults (11-30 years), corresponding to reproductive age. In contrast, abdominal and pelvic pain was the leading complaint among middle-aged and older adults (31-90 years), likely linked to gastrointestinal, gynecological, or urological conditions.

Trauma-related cases comprised 27.5% of all diagnoses, with extremity injuries (10.5%), polytrauma (6.2%), neck and head injuries (4%), and falls (3%) being the most common. These diagnoses were significantly more frequent on weekends, with over 50% occurring on Sundays. This pattern may reflect a patient population composed mainly of working-class individuals engaged in manual labor, as our hospital provides social services for uninsured patients. Additionally, weekend leisure activities and increased time spent at home may elevate the risk of injuries, particularly among vulnerable groups. Similar trends have been reported in other studies, such as one conducted at a trauma center in Iran, where motor vehicle accidents (39.5%) were the most frequent trauma mechanism, followed by falls (30.2%) [27]. In India, motor vehicle accidents accounted for 74.2% of trauma cases, with falls comprising 10.2%; extremity and head injuries were the most common diagnoses [28].

Gunshot wounds (GSWs) and stab wounds (SWs) are prevalent injuries in Mexico. According to INEGI, in 2022, assaults involving these weapons were the eighth leading cause of death nationwide [29]. In our study, GSWs accounted for 1.5% of all emergencies, while SWs made up 0.8%. In contrast, data from the United States National Trauma Data Bank reported 32.1% GSWs and 1.97% SWs [30]. Turkey reported 8.1% of injuries as SWs and GSWs over a full year [31]. The lower prevalence of these injuries in our population correlates with regional crime rates. In 2023, Nuevo León, where Monterrey is located, had a crime rate of 28,171 per 100,000 inhabitants, lower than the national average of 33,267 per 100,000. The predominant crimes were robbery and public assault [32]. Nuevo León’s economic stability, being the third-highest GDP state in Mexico in 2022 [33], likely contributes to a lower incidence of violent injuries.

A study on alcohol consumption in Monterrey found that the highest consumers were men aged 31-45, followed by those aged 18-30 [34]. This aligns with the demographic of emergency department users, where the average age was 39 years, and the most represented age group was 21-30 years (24%, 3,541 patients). The study also reported high rates of concurrent alcohol and tobacco use, substances associated with increased non-communicable disease incidence. Further research integrating socioeconomic and mental health factors could provide deeper insights into the relationship between alcohol consumption and emergency department utilization.

The busiest days for emergencies were Saturdays and Mondays, accounting for 15.2% and 15% of visits, respectively. This pattern aligns with studies from Barcelona and Peru, where Mondays saw the highest emergency department usage [8,35]. However, the Barcelona study observed a steady decline in cases throughout the week, reaching its lowest on Saturday before increasing again on Sunday. The increased demand on Saturdays and Mondays may be linked to cultural habits, with weekend recreational activities and alcohol consumption elevating health risks. Additionally, work-related constraints may lead patients to delay seeking medical attention until the start of the workweek [36]. Further investigation is needed to understand these sociocultural factors, and community awareness programs could encourage timely medical assistance and reduce delays in care [37].

Most patients were admitted through internal medicine, followed by general surgery, gynecology and obstetrics, and traumatology and orthopedics. A study in Sri Lanka at a tertiary care hospital showed similar trends, with pediatrics also being a major specialty [9]. Likewise, findings from Ethiopia indicated a predominance of medical emergencies, followed by traumatic and surgical cases [38]. These trends align with our study’s most common diagnoses, as abdominal/pelvic pain is managed by internal medicine or general surgery, pregnancy by gynecology and obstetrics, and extremity injuries by general surgery or orthopedics.

This study has limitations. The cross-sectional design provides only a snapshot of data, limiting causal inferences. The four-month study period does not account for seasonal variations. Additionally, variables such as trauma mechanisms (e.g., motor vehicle accidents, and violence), toxicology (alcohol and drug use), resuscitation needs, wait times, and emergency department length of stay were not included. Reliance on medical and nursing staff for data collection may introduce reporting bias.

As one of the few studies on emergency distribution in Mexico, these findings can guide infrastructure and personnel allocation in tertiary hospitals in cities with similar characteristics to Monterrey. The similarity in emergency distribution across developing areas suggests that findings from developed nations may not always be applicable. Continued evaluation of emergency characteristics, particularly time trends and diagnoses, could inform targeted healthcare interventions. Improved resource allocation and staffing adjustments based on peak hours could enhance patient care and reduce wait times. Regular data audits could further optimize hospital function and adaptation to patient needs. Future research should systematically compare data from other regions to contextualize findings and assess external validity.

## Conclusions

This study shows that the majority of patients in the emergency department are female, aged 21-30 years, and from the metropolitan area of Monterrey. Throughout the week, the highest patient influx occurs on Saturdays, with peak times at 1:00 p.m. and 10:00 p.m. Nontraumatic emergencies are the most common, with abdominal/pelvic pain being the leading diagnosis. Among traumatic cases, extremity injuries are the most frequent. The internal medicine service handles the majority of cases, while traumatic emergencies are primarily managed by surgical specialties, such as general surgery and orthopedics.

## References

[1] (2022). Estrategia Nacional De Salud: Para Los Objetivos Sanitarios Al 2030. https://cens.cl/wp-content/uploads/2022/03/Estrategia-Nacional-de-Salud-al-2030.pdf.

[2] Ebner M, Gaete T, Guzman P (2016). Epidemiological characterization of attention in a primary attention and emergency service of the metropolitan region, Chile 2015. Rev ANACEM.

[3] Bonatti H, Calland JF (2008). Trauma. Emerg Med Clin North Am.

[4] (2024). Accidentes de tránsito. https://www.inegi.org.mx/temas/accidentes/.

[5] Paudel S, Dhungana S, Pokhrel N, Dhakal GR (2021). Epidemiology of trauma patients presented at emergency department of trauma center. J Nepal Health Res Counc.

[6] Collie A, Simpson PM, Cameron PA (2019). Patterns and predictors of return to work after major trauma: a prospective, population-based registry study. Ann Surg.

[7] Hitzek J, Fischer-Rosinský A, Möckel M, Kuhlmann SL, Slagman A (2022). Influence of weekday and seasonal trends on urgency and in-hospital mortality of emergency department patients. Front Public Health.

[8] Torné Vilagrasa E, Guarga Rojas A, Torras Boatella MG, Pozuelo García A, Pasarin Rua M, Borrell Thió C (2003). Analysis of demand in the emergency services of Barcelona [Article in Spanish]. Aten Primaria.

[9] Jayasekera P, Dassanayake G, Bandara K, Jayawardhena L, Malkanthi KM (2020). A study of the pattern of admissions to the accident and emergency (A&E) department of a tertiary care hospital in Sri Lanka. Emerg Med Int.

[10] Wai AK, Chor CM, Lee AT, Sittambunka Y, Graham CA, Rainer TH (2009). Analysis of trends in emergency department attendances, hospital admissions and medical staffing in a Hong Kong university hospital: 5-year study. Int J Emerg Med.

[11] Dresden SM (2013). Measuring the value of the emergency department from the patient's perspective. Ann Emerg Med.

[12] Marengoni A, Angleman S, Melis R (2011). Aging with multimorbidity: a systematic review of the literature. Ageing Res Rev.

[13] Barnett B, Mercer SW, Norbury M, Watt G, Wyke S, Guthrie B (2012). Epidemiology of multimorbidity and implications for health care, research, and medical education: a cross-sectional study. Lancet.

[14] Galarraga JE, Pines JM (2016). Costs of ED episodes of care in the United States. Am J Emerg Med.

[15] (2023). La situación demográfica de México 2023. https://www.gob.mx/conapo/documentos/la-situacion-demografica-de-mexico-2023.

[16] The best-equipped hospitals in Latin America in 2023. https://globalhealthintelligence.com/the-best-equipped-hospitals-in-latin-america-in-2023/.

[17] Arreola-Risa C, Mock CN, Padilla D, Cavazos L, Maier RV, Jurkovich GJ (1995). Trauma care systems in urban Latin America: the priorities should be prehospital and emergency room management. J Trauma.

[18] Velázques-Guzmán M, Morales-Hernábndez A, Fonseca-Carrillo I (2017). Clinical correlation of triage with clinical diagnosis at entering and discharge in patients assisting to emergency room of a private hospital. Med Int Mex.

[19] Aylin P, Yunus A, Bottle A, Majeed A, Bell D (2010). Weekend mortality for emergency admissions. A large, multicentre study. Qual Saf Health Care.

[20] Vashi AA, Urech T, Carr B, Greene L, Warsavage T Jr, Hsia R, Asch SM (2019). Identification of emergency care-sensitive conditions and characteristics of emergency department utilization. JAMA Netw Open.

[21] Escobedo F, González-Gil L, Salarchis M, Manzano A, López J, Albaladejo C (1997). Evaluation of patients attending hospital casualty who came from a particular health district. Aten Primaria.

[22] Çıkrıkçı Işık G, Tandoğan M, Şafak T, Çevik Y (2020). Retrospective analyses of frequent emergency department users. Eurasian J Emerg Med.

[23] Cairns C, Ashman JJ, Kang K (2024). Emergency department visit rates by selected characteristics: United States, 2022. NCHS Data Brief.

[24] Martín-Sánchez FJ, Fernández Alonso C, Merino C (2010). The geriatric patient in the emergency department [Article in Spanish]. An Sist Sanit Navar.

[25] (2025). Pobreza y personas mayores en México 2020. https://www.coneval.org.mx/Medicion/MP/Paginas/Pobreza_Personas_Mayores.aspx#:~:text=De%202016%20a%202018%2C%20el,puntos%20porcentuales%2C%20respecto%20a%202018.

[26] Vilagrasaa ET, Rojasa AG, Boatellaa MT, Garcíaa AP, Ruab MP, Thiób CB (2003). Analysis of demand in emergency services in Barcelona [Article in Spanish]. Aten Primaria.

[27] Roshanaei G, Khoshravesh S, Abdolmaleki S, Bathaei T, Farzian M, Saatian M (2022). Epidemiological pattern of trauma patients based on the mechanisms of trauma: trends of a regional trauma center in Midwest of Iran. BMC Emerg Med.

[28] Jain M, Radhakrishnan RV, Mohanty CR (2020). Clinicoepidemiological profile of trauma patients admitting to the emergency department of a tertiary care hospital in eastern India. J Family Med Prim Care.

[29] (2025). Estadísticas De Defunciones Registradas (EDR) 2022. https://www.inegi.org.mx/contenidos/saladeprensa/boletines/2023/EDR/EDR2022.pdf.

[30] Zeineddin A, Williams M, Nonez H (2021). Gunshot injuries in american trauma centers: analysis of the lethality of multiple gunshot wounds. Am Surg.

[31] Sogut O, Sayhan MB, Gokdemir MT (2011). Analysis of hospital mortality and epidemiology in trauma patients: a multi-center study. J Curr Surg.

[32] (2025). Incidencia delictiva. https://www.inegi.org.mx/temas/incidencia/.

[33] (2025). Producto Interno Bruto por Entidad Federativa (PIBE). https://www.inegi.org.mx/contenidos/saladeprensa/boletines/2023/PIBEF/PIBEF2022.pdf.

[34] Ruiz-Juan F, Isorna-Folgar M, Vaquero-Cristóbal R, Ruiz-Risueño J (2016). Alcohol consumption in adults from Monterrey: relationship with physical-sports activity and family [Article in Spanish]. Nutr Hosp.

[35] Taype-Huamaní Waldo, Chucas-Ascencio Luis, De la Cruz-Rojas Lucila, Amado-Tineo Jose (2019). Waiting time for urgent medical care in a tertiary hospital after implementing a process improvement program [Article in Spanish]. An Fac Med.

[36] López-García E, Pérez-López C, Postigo C (2020). Assessing alcohol consumption through wastewater-based epidemiology: Spain as a case study. Drug Alcohol Depend.

[37] Lauver D (1992). A theory of care-seeking behavior. Image J Nurs Sch.

[38] Woyessa AH, Dibaba BY, Hirko GF, Palanichamy T (2019). Spectrum, pattern, and clinical outcomes of adult emergency department admissions in selected hospitals of Western Ethiopia: a hospital-based prospective study. Emerg Med Int.

